# WWOX sensitises ovarian cancer cells to paclitaxel via modulation of the ER stress response

**DOI:** 10.1038/cddis.2017.346

**Published:** 2017-07-27

**Authors:** Szymon Janczar, Jaya Nautiyal, Yi Xiao, Edward Curry, Mingjun Sun, Elisa Zanini, Adam JW Paige, Hani Gabra

**Affiliations:** 1 Department of Surgery and Cancer, Molecular Therapeutics Unit and Ovarian Cancer Action Research Centre, Imperial College London, Hammersmith Hospital, London, UK; 2 Department of Pediatrics, Oncology, Hematology and Diabetology, Medical University of Lodz, Lodz, Poland; 3 Department of Life Sciences, University of Bedfordshire, Luton, UK; 4 Clinical Discovery Unit, Early Clinical Development, AstraZeneca, Cambridge, UK

## Abstract

There are clear gaps in our understanding of genes and pathways through which cancer cells facilitate survival strategies as they become chemoresistant. Paclitaxel is used in the treatment of many cancers, but development of drug resistance is common. Along with being an antimitotic agent paclitaxel also activates endoplasmic reticulum (ER) stress. Here, we examine the role of *WWOX* (WW domain containing oxidoreductase), a gene frequently lost in several cancers, in mediating paclitaxel response. We examine the ER stress-mediated apoptotic response to paclitaxel in WWOX-transfected epithelial ovarian cancer (EOC) cells and following siRNA knockdown of WWOX. We show that WWOX-induced apoptosis following exposure of EOC cells to paclitaxel is related to ER stress and independent of the antimitotic action of taxanes. The apoptotic response to ER stress induced by WWOX re-expression could be reversed by WWOX siRNA in EOC cells. We report that paclitaxel treatment activates both the IRE-1 and PERK kinases and that the increase in paclitaxel-mediated cell death through WWOX is dependent on active ER stress pathway. Log-rank analysis of overall survival (OS) and progression-free survival (PFS) in two prominent EOC microarray data sets (Tothill and The Cancer Genome Atlas), encompassing ~800 patients in total, confirmed clinical relevance to our findings. High WWOX mRNA expression predicted longer OS and PFS in patients treated with paclitaxel, but not in patients who were treated with only cisplatin. The association of WWOX and survival was dependent on the expression level of glucose-related protein 78 (GRP78), a key ER stress marker in paclitaxel-treated patients. We conclude that WWOX sensitises EOC to paclitaxel via ER stress-induced apoptosis, and predicts clinical outcome in patients. Thus, ER stress response mechanisms could be targeted to overcome chemoresistance in cancer.

Epithelial ovarian cancer is the most lethal gynaecological malignancy. Use of platinum and taxane-based chemotherapy result in high response rates, but 70% of patients relapse and develop drug-resistant disease.^1^ Paclitaxel stabilises microtubule assembly, resulting in a mitotic block of cell cycle leading to apoptosis.^2^ The cytotoxic effects of taxanes are not just because of its antimitotic function^3^ but are, in part, mediated by endoplasmic reticulum (ER) stress/unfolded protein response (UPR).^4, 5, 6, 7, 8^ UPR is a programme initiated by the accumulation of unfolded proteins in ER to re-establish homeostasis by activation of chaperones and translation inhibition.^9^

The WWOX (WW domain containing oxidoreductase) gene on chromosome 16q23-24 is located at the same locus as the common fragile site FRA16D.^10^ WWOX loss increases tumour susceptibility in several mouse models.^11, 12, 13^ WWOX expression is lost or downregulated in most cancers because of genomic disruption or epigenetic silencing, and recently The Cancer Genome Atlas data sets have highlighted 44 novel somatic mutations in WWOX in various cancer types, several of which lead to changes in the protein function.^14, 15, 16, 17, 18^ WWOX is highly expressed in secretory epithelia, in reproductive, exocrine and endocrine organs and also in neuronal bodies throughout the central nervous system. Mutations in WWOX have been reported in several neurological pathologies.^18, 19^

WWOX is lost in 30% of ovarian carcinomas and this is associated with disease progression, and poor prognosis.^20^ We previously demonstrated that WWOX transfection of PEO1 ovarian cancer cells abolished their *in vivo* tumorigenicity because of altered interaction of tumour cells with surrounding ECM.^21^ This did not correlate with decreased *in vitro* growth or survival, but was as a result of reduced integrin *α*3 levels.^21^ There was also no effect of WWOX on apoptosis following cisplatin exposure.^21^ As standard chemotherapy treatment in ovarian cancer is a combination of platinum and paclitaxel, we examined the impact of WWOX transfection on paclitaxel response. We report that WWOX transfection into the non-expressing PEO1 ovarian cancer cell line^21, 22^ causes sensitisation to paclitaxel, as demonstrated by increased apoptosis following exposure with paclitaxel. This is independent of the antimitotic function of taxanes, but is related to apoptosis because of ER stress induced by paclitaxel. To mimic the natural course of events involving WWOX loss in cancer, we used siRNA knockdown of endogenous WWOX to study its role in paclitaxel-induced cell death in SKOV-3 and OVCAR-4 cell lines. We conclude that (1) both IRE-1 and PERK arms of UPR get activated on exposure of ovarian cancer cells to paclitaxel and active ER stress is a requirement for WWOX-mediated cell death caused by paclitaxel and (2) WWOX expression may predict patient outcome to taxane-based chemotherapy.

## Results

### WWOX sensitises ovarian cancer cells to paclitaxel

**Figure 1:**
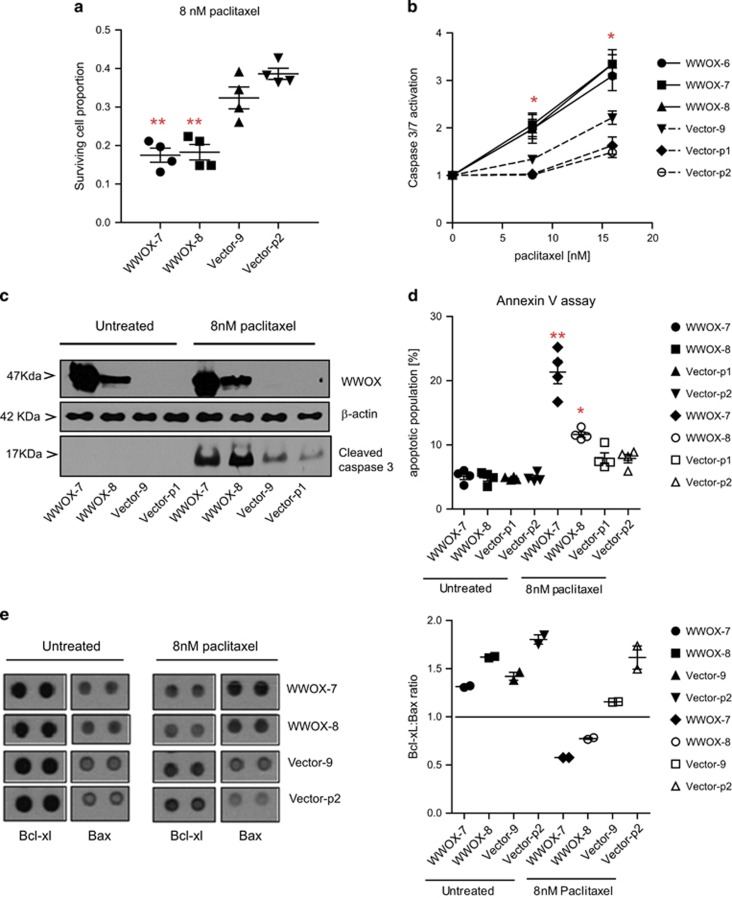
WWOX-transfected PEO1 lines show increased apoptosis following treatment with paclitaxel, compared with vector-transfected lines. (**a**) Relative cell survival following treatment with paclitaxel for 72 h, measured by SRB assay. Means of quadruplicate experiments±S.E.M. (**b**) Caspase-3/7 activation following treatment with paclitaxel for 24 h, measured by CaspaseGlo3/7 reagent (Promega). Means of six experiments±S.E.M. (**c**) Immunoblot showing activated caspase-3 levels (MAB835 antibody; R&D Systems) following treatment with 8 nM of paclitaxel for 24 h. *β*-Actin bands (AC15 antibody; Sigma) show equal loading of blot and WWOX immunoblot (antibody gifted by Dr. M Aldaz, MD Anderson Cancer Centre) demonstrates successful knockdown. (**d**) Early apoptotic (Annexin V-positive/propidium iodide (PI)-negative) cells following treatment with paclitaxel for 24 h. Means of quadruplicate experiments±S.E.M. (**e**) Left panel: Bcl-xL and Bax probe spots from protein arrays, showing increased Bax and decreased Bcl-xL in WWOX-transfected lines following exposure with paclitaxel for 24 h; right panel: densitometry from protein array shows lower Bcl-xL:Bax ratio in WWOX-transfected cells treated with paclitaxel. Means of two probe spots±S.D. (*P<0.05; **P<0.01)

WWOX reconstitution in PEO1 ovarian cancer cells (WWOX-7, WWOX-8) significantly enhanced paclitaxel-mediated cell death (Figures 1a, *P*<0.01) compared with vector-transfected control cells (Vector-9, Vector-p2). We examined caspase activation, Annexin V positivity and an antibody array to measure the relative levels of apoptosis-related proteins in these cell lines. WWOX transfection significantly increased caspase-3/7 activation following exposure with paclitaxel 24 h at either 8 or 16 nM, as compared with the vector-transfected control lines (Figures 1b, *P*<0.05). Increased levels of cleaved caspase-3 was observed in WWOX-transfected lines on treatment with paclitaxel (Figure 1c). Following paclitaxel exposure, WWOX-transfected cells also displayed significantly higher levels of early apoptotic cells (Annexin V-positive, PI-negative) (Figures 1d, *P*<0.05). The antibody array demonstrated no consistent differences in the expression of apoptosis-related proteins in basal conditions, but upon paclitaxel exposure, a lower B-cell lymphoma-extra large (Bcl-xL)/Bcl-2-associated x (Bax) ratio in WWOX-transfected cells was observed (Figure 1e).

**Figure 2:**
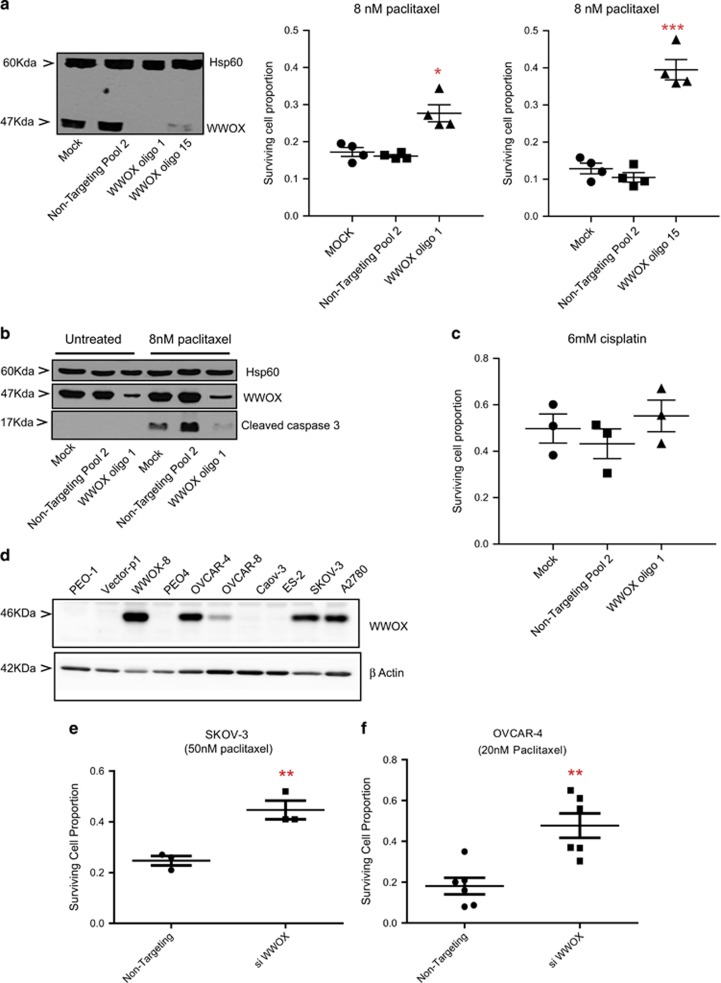
Knockdown of WWOX in WWOX-transfected PEO1, SKOV-3 and OVCAR-4 cells decreases apoptosis following treatment with paclitaxel compared with non-targeting controls. (**a**) Relative cell survival following treatment with paclitaxel for 72 h, measured by SRB assay. Means of quadruplicate experiments±S.E.M. Western blot shows loss of WWOX expression after treatment with WWOX siRNA for 72 h, as compared with mock-transfected and non-targeting cells in PEO1 cells. (**b**) Immunoblot showing activated caspase-3 levels (MAB835 antibody; R&D Systems) following treatment with 8 nM of paclitaxel for 24 h. Hsp60 (ab46798 antibody; abcam) bands show equal loading of blot, while WWOX blot shows successful knockdown in PEO1 cells. (**c**) Relative cell survival following treatment with cisplatin for 72 h, measured by SRB assay. Means of triplicate experiments±S.E.M. (*P<0.05; ***P<0.005). (**d**) Immunoblot showing endogenous WWOX expression (ab137726 antibody; abcam) in ovarian cancer cell lines. *β*-Actin (no. 4970; Cell Signalling Technology) has been used as a loading control. Relative cell survival 72 h after treatment with siWWOX or non-targetting control and 48 h after treatment with 50 and 20 nM paclitaxel in SKOV-3 (**e**) and OVCAR-4 (**f**) cells, respectively

Two independent siRNA oligos were used to knockdown WWOX in WWOX-8 cell line. This decreased the apoptotic response to these cells to 8 and 16 nM paclitaxel (not shown) as compared with control transfections (Figures 2a, *P<0.05 and ***P<0.005) and cleaved caspase-3 expression (Figure 2b), confirming that paclitaxel chemoresponse is influenced by WWOX expression. In parallel, WWOX status has no impact on cisplatin response (Figure 2c) to complement the previous data generated with stable transfectants.^21^ To mimic the loss/reduction of WWOX as it happens in the course of cancer, we knocked down endogenous WWOX in ovarian cancer cells to monitor the impact on cell survival. We screened several ovarian cancer cells to detect WWOX expression (Figure 2d) and found endogenous protein expression in OVCAR-4, OVCAR-8, SKOV-3 and A2780 cell lines (Figure 2d). siRNA knockdown of WWOX in OVCAR-4 and SKOV-3 cell lines led to (Figure 5b) an improvement in cell survival in both SKOV-3 (Figure 2e,*P*=0.0082) and OVCAR-4 cell lines (Figure 2f, *P*=0.0022) on treatment with 50 and 20 nM paclitaxel, respectively.

### WWOX-driven differential response to paclitaxel is independent of the antimitotic action of taxanes, integrin *α*3 and transforming growth factor-*β*1 levels

**Figure 3:**
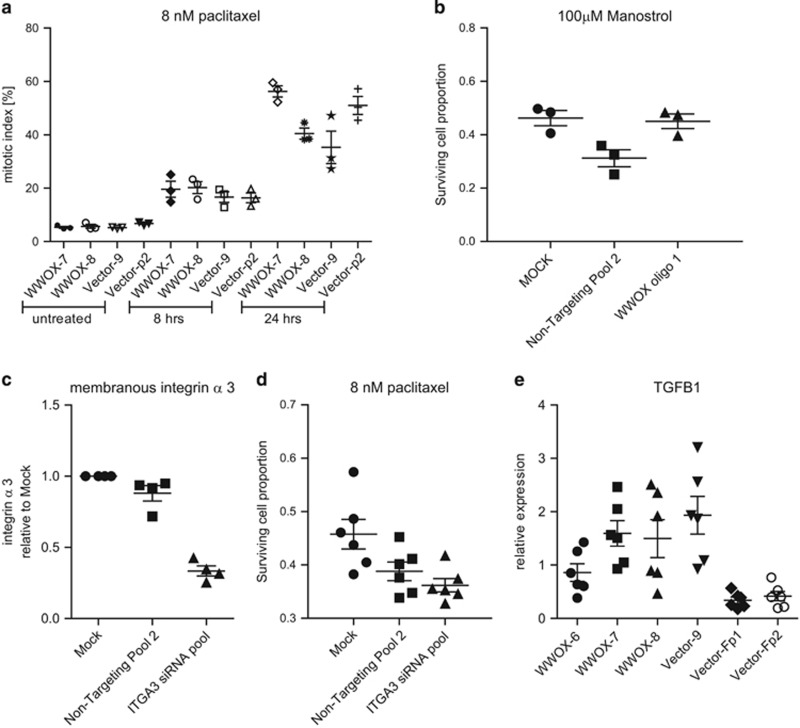
The differential response of WWOX-transfected cells to paclitaxel is not related to the antimitotic action of taxanes, and WWOX-induced apoptosis is independent of ITGA3 and TGFBI. (**a**) WWOX transfection does not alter the percentage of mitotic (MPM2 antibody-labelled (Millipore)) cells as determined by FACS following treatment with paclitaxel. Means of triplicate experiments±S.E.M. (**b**) WWOX depletion does not alter the cytotoxic response to monastrol. Cell survival following treatment with monastrol for 72 h, measured by SRB assay. Means of triplicate experiments±S.E.M. (**c**) ITGA3 expression following siRNA knockdown as measured by FACS, compared with mock-transfected and non-targeting siRNA. Means of quadruplicate experiments±S.E.M. (**d**) ITGA3 siRNA knockdown did not affect cell survival following exposure with 8 nM paclitaxel, measured by SRB assay. Means of six experiments±S.E.M. (**e**) TGFBI mRNA expression is not related to WWOX expression in WWOX- and vector-transfected PEO1 lines. Means of six experiments±S.E.M. NT, non-targeting siRNA

We next examined mitotic arrest in paclitaxel-treated cells to determine whether reduced survival following WWOX overexpression was due to an increased proportion of cells arrested in mitosis (Figure 3a). WWOX status had no impact on the response of PEO1 cells to the kinesin Eg5 inhibitor monastrol, a selective antimitotic agent^23^ (Figure 3b). Thus, the effect of WWOX on paclitaxel response is distinct from the antimitotic function of taxanes. We hypothesised that a differential response to paclitaxel treatment following WWOX transfection might be due to ITGA3 regulation.^21^ However, we observed no significant impact of ITGA3 knockdown in PEO1 cell line on responses to paclitaxel (Figures 3c and d). We examined the the levels of transforming growth factor-β1 (TGFB1), which has previously been shown to be a critical mediator of paclitaxel response in ovarian cancer.^24^ We could not detect measurable levels of secreted TFGB1 in culture medium in PEO1 clones, but quantitative RT-PCR (QPCR) revealed variable TGFBI levels and no correlation with WWOX expression (Figure 3e).

### Paclitaxel induces ER stress response and WWOX determines cell fate in response to prolonged ER stress

**Figure 4:**
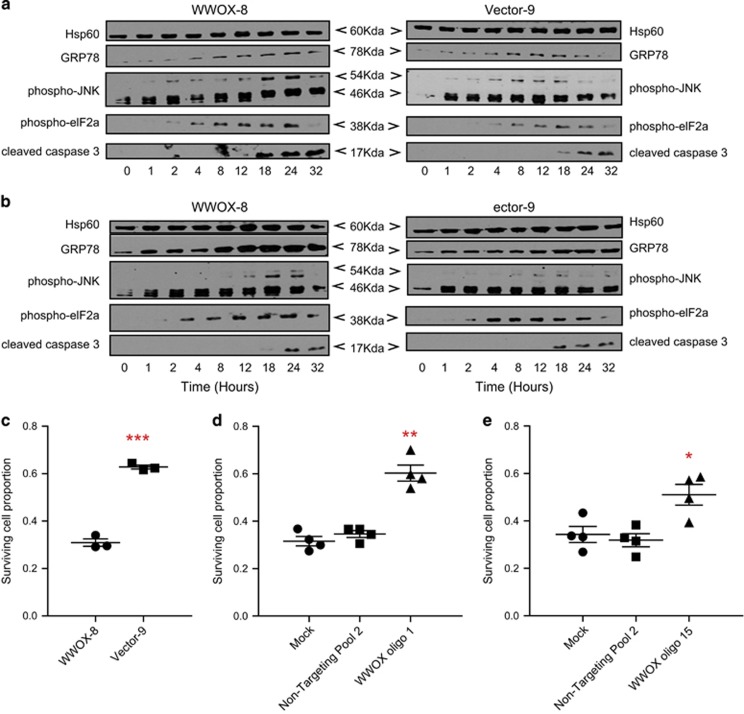
Paclitaxel evokes ER stress in PEO1 cells and WWOX sensitises PEO1 cells to the ER stress inducer tunicamycin. (**a**) WWOX-8 and control Vector-9 cells were treated with 8 nM paclitaxel or (**b**) 200 ng/*μ*l tunicamycin. Floating and adherent cells were lysed at 0, 1, 2, 4, 8, 12, 18, 24 and 32 h time points and analysed by western blotting. (**c**) Relative cell survival following treatment with tunicamycin for 48 h, measured by SRB assay. Means of triplicate experiments±S.E.M. (**d** and **e**) Knockdown of WWOX in WWOX-8 cells decreases apoptosis following treatment with tunicamycin, compared with mock-transfected or non-targeting controls. Relative cell survival following exposure with tunicamycin for 48 h, measured by SRB assay. Means of quadruplicate experiments±S.E.M. (*P<0.05; **P<0.01; ***P<0.005). NT, non-targeting siRNA

Treatment of PEO1 cells with 8 nM paclitaxel led to induction of ER stress, as indicated by upregulation of GRP78 chaperone and phosphorylation of eukaryotic translation initiation factor 2A (eIF2A), as well as c-Jun N-terminal kinase (JNK) and p38 mitogen-associated protein kinase (MAPK) activation^25^ (Figure 4a and Supplementary Figure 1). Similar changes were observed in a parallel time-course experiment following exposure to tunicamycin, a classic inducer of ER stress^25^ (Figure 4b). WWOX-transfected cells displayed significantly reduced survival rates when exposed to tunicamycin (200 ng/*μ*l) (Figure 4c, *P*<0.005), whereas WWOX siRNA knockdown increased survival (Figures 4d, *P*<0.01 and Figure 4e,*P*<0.05). Thus, WWOX expression directs UPR induced by tunicamycin or paclitaxel towards an apoptotic outcome. WWOX-dependent apoptosis during ER stress was examined in context of MAPK signalling.^26^ Inhibition of JNK^27^ abrogated paclitaxel response in WWOX-8 cells (Supplementary Figure 2) but marginally prevented tunicamycin toxicity (Supplementary Figure 3). Inhibition of JNK quite remarkably antagonised the antimitotic (Supplementary Figure 4) function of taxanes in PEO1 cells. We found no evidence of WWOX-JNK1 interaction in co-immunoprecipitation experiments following exposure with paclitaxel (Supplementary Figure 5 and not shown).

**Figure 5:**
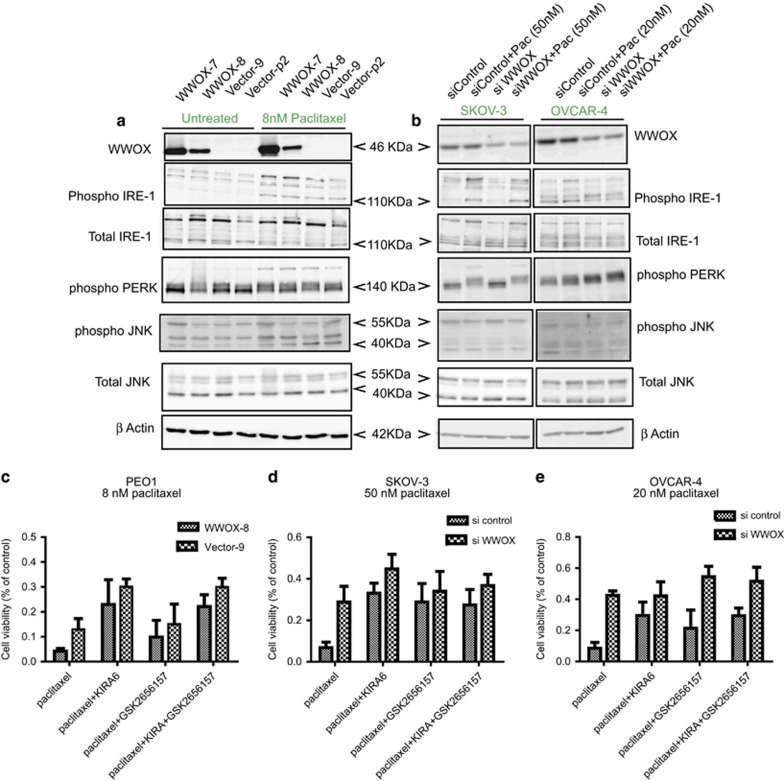
WWOX regulates ER stress through IRE-1 and PERK. Western blots showing the expression of WWOX and ER stress regulators IRE-1 (ab48187 and ab37073 antibodies; abcam), PERK (no. 3192; Cell Signalling Technology), JNK (MAB2076 (Total) and AF1205 (phospho) antibodies; R&D Systems) (**a**) untreated or treated with 8 nM paclitaxel for 24 h in PEO1 cell lines in the presence or absence of WWOX. (**b**) SKOV-3 and OVCAR-4 cells, 72 h after treatment with siWWOX or no targeting control and 24 h after treatment with paclitaxel in 50 nM SKOV-3 and 20 nM OVCAR-4 cells. *β*-Actin has been used as a loading control. Cell viability was measured through SRB assay in (**c**) WWOX-8 and Vector-9 (**d**) SKOV-3 (72 h siRNA treatment) and (**e**) OVCAR-4 cells (72 h siRNA treatment) with IRE-1 inhibitor (KIRA6; 532281; Calbiochem) and PERK (GSK2656157; S7033; Selleckchem) alone or together in combination with paclitaxel. Cells were treated with 1 *μ*g/μl inhibitors 2 h before paclitaxel treatment for 24 h. Triplicate experiments were performed

To investigate ER stress induced by paclitaxel and the role of WWOX in this context, we carried out analysis of (1) PEO1 cells (Figures 5a) and (2) by knocking down endogenous WWOX expression in SKOV-3 and OVCAR-4 cell lines (Figure 5b). We examined the expression of the two key ER transmembrane kinases IRE-1 and PERK, which activate ER stress responses. Both IRE-1 and PERK showed induction or increase of phosphorylation under the influence of paclitaxel in all cell lines compared with untreated cells. When probed with a phosphorylated IRE-1 antibody, a band at 110 kDa appeared in paclitaxel-treated cells. The identity of this band was confirmed using another antibody detecting total IRE-1 protein (Figures 5a and b). In a control treatment of the cells with thapsigargin (a strong inducer of ER stress), a slightly upward shifted pIRE-1 band was observed (Supplementary Figure 6), suggesting differences in the phosphorylation status of the protein under paclitaxel and thapsigargin treatments. It was noteworthy, however, that the intensity of the pIRE-1 band induced by thapsigargin was less in WWOX-null Vector-9 compared with WWOX-overexpressing WWOX-8 cells indicating higher levels of ER stress in the latter (Supplementary Figure 6). PERK expression showed a shift from a distinct lower band in untreated conditions to a characteristic fuzzy upward shifted band/smear after paclitaxel treatment (Figures5a and Figures5b), indicating activation of PERK.^28, 29^ A similar response was observed on treating the PEO1 cells with thapsigargin and analysing PERK expression with a different antibody (abcam, Cambridge, UK; ab65142). No changes in activation of PERK were observed as a result of WWOX status (Figures 5a and b and Supplementary Figure 6). Changes in the phosphorylated status of JNK1/2 in the PEO1 cells with WWOX overexpression were observed, but this was not pronounced in SKOV-3 and OVCAR-4 cells with WWOX knockdown. To examine upstream events in the ER stress-mediated apoptosis analysis of expression of procaspase and cleaved caspase-2 and -7 was performed. Increase in the expression of cleaved caspase-7 in paclitaxel-treated cells (Supplementary Figure 7) was observed, but this was not influenced by WWOX expression. Subtle increase in the expression of cleaved caspase-2 in paclitaxel-treated WWOX-overexpressing PEO cells and a reduction in SKOV-3 and OVCAR-4 cells on siWWOX was observed (Supplementary Figure 7).

Since the adaptive and maladaptive arms of UPR coexist under ER stress, it is essential to determine which arm is dominant in paclitaxel-induced ER stress and how these are affected by WWOX expression. Thus, cells were treated with paclitaxel alone or in combination with IRE-1 inhibitor KIRA6 and/or PERK inhibitor GSK2656157 and cell viability was analysed. As observed in the earlier results (Figures 1a and Figures 2e, Figures 2f), WWOX expression sensitised all three cell lines, that is, PEO1 (Figure 5c), SKOV-3 (Figure 5d) and OVCAR-4 cells (Figure 5e) to paclitaxel-induced cell death. Cotreatment of cells with KIRA6 and paclitaxel improved cell viability compared with the paclitaxel-alone group in all cell lines (Figures 5c–e). Combination of PERK inhibitor, GSK2656157, with paclitaxel enhanced cell survival in only SKOV-3 cells (Figure 5d). Notably, in the absence of WWOX in PEO1 cells (Vector-9) or when WWOX expression was knocked down by siRNA in SKOV-3 and OVCAR-4, cells were less sensitive to KIRA6 and GSK2656157 as shown by the blunted effects on cell viability (Figures 5c–e) compared with the respective paclitaxel-alone treated groups. Among the combination treatments, the visible rescue in cell viability on WWOX knockdown was seen in GSK2656157-treated OVCAR-4 cells, indicating that WWOX may not be acting by regulating cell survival through the PERK pathway in this cell line (Figure 5e). To further examine the impact of the IRE-1 and PERK inhibitors with paclitaxel on cell survival, we carried out propidium podide (PI) staining in OVCAR-4 and SKOV-3 cells treated with paclitaxel alone or in combination with KIRA6 and GSK2656157. Combination of IRE-1 inhibitor KIRA6 with paclitaxel reduced cell death, while combination with the PERK inhibitor GSK2656157 led to no change or slight increase in cell death (Supplementary Figure 8), indicating that the two pathways might have different roles in mediating the response to paclitaxel in these ovarian cancer cell lines (Figure 5 and Supplementary Figure 8).

### WWOX expression predicts PFS in paclitaxel-treated ovarian cancer patients

We used data from two large cohorts of primary ovarian tumours to investigate the associations between WWOX expression and clinical outcome to chemotherapy. The data set referred to as ‘TCGA’, featuring over 500 primary tumour samples, was obtained from The Cancer Genome Atlas (https://tcga-data.nci.nih.gov/tcga/)^30^ The data set referred to as ‘Tothill’, featuring nearly 300 primary tumour samples, was obtained from Gene Expression Omnibus (series accession GSE9891).^31^ Raw gene expression data (Affymetrix CEL files) were obtained for each data set and normalised using robust multiarray average (RMA).^32^

#### WWOX expression and OS

**Figure 6:**
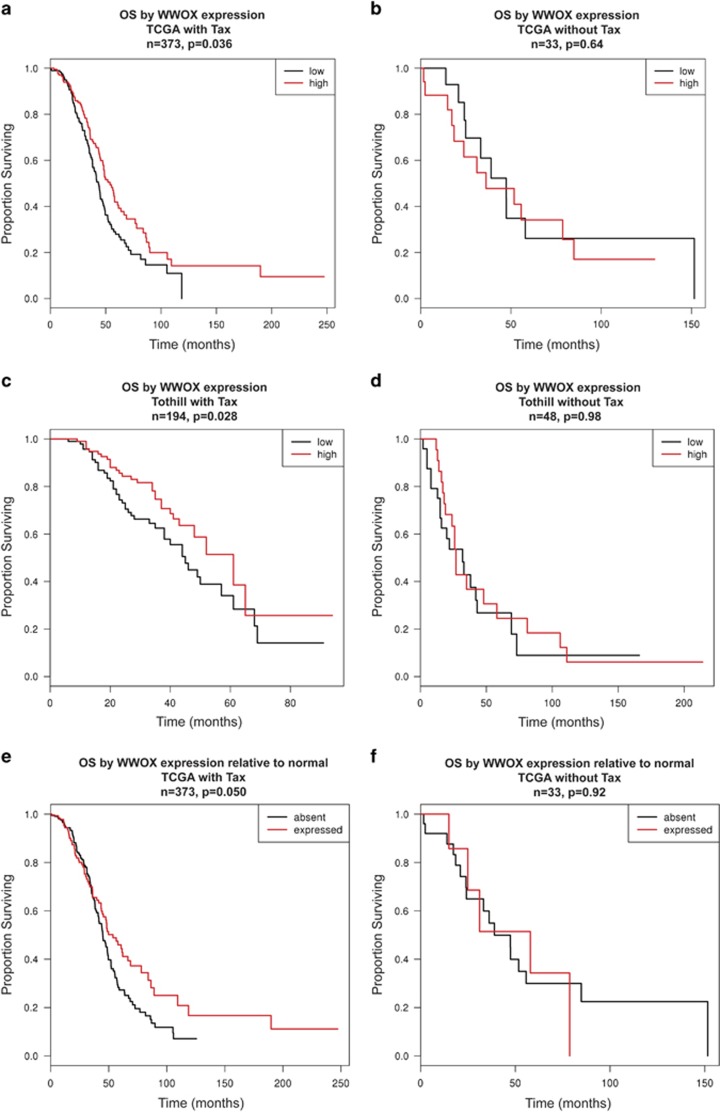
WWOX levels predict OS in taxane-treated ovarian cancer patients. Kaplan–Meier plots to show the effect of WWOX expression on OS in two ovarian cancer cohorts, namely (**a**) TCGA (*n*=491) and (**b**) Tothill (*n*=258). OS plots of patients in the TCGA cohort treated with platinum alone (**d**) and taxane+platinum (**c**) and survival plots in the Tothill data set of patients treated with platinum alone (**f**) or taxane+platinum (**e**). Red line represents patients with higher WWOX expression, whereas black line represents patients with lower WWOX expression. (**g** and **h**) OS curves when the TCGA data set was analysed, setting a cutoff to reflect the absence or presence of WWOX in patients who received platinum treatment (**h**) or platinum in combination to paclitaxel (**g**). Black represents WWOX absence, whereas red line represents WWOX expression

Fitting Cox proportional hazards regression models revealed significant association between higher WWOX expression and longer OS in patients treated with platinum in combination with taxane in both TCGA (*n*=373, hazard ratio=0.48, *P*=0.036) and Tothill (*n*=194, hazard ratio=0.53, *P*=0.028) cohorts. In both the cohorts higher expression of WWOX was associated with longer survival times even when the extent of disease after primary surgery was taken into account (TCGA hazard ratio=0.48, *P*=0.046; Tothill hazard ratio=0.67, *P*=0.19), implying that WWOX levels had a clear impact on patient survival. When the patients who received taxanes in the first-line chemotherapy are excluded, the association between WWOX expression and OS was lost (TCGA *n*=33, hazard ratio=1.9, *P*=0.64; Tothill *n*=48, hazard ratio=1.0, *P*=0.98). To illustrate these associations, Figures 6a and c show Kaplan–Meier plots of OS when patients are partitioned into WWOX-high (greater than median expression) and WWOX-low subsets, for the cohorts receiving both platinum and taxane. *P*-values reported in the figures represent results from log-rank test based on dichotomisation used to draw curves. Figures 6b and d show equivalent Kaplan–Meier plots of OS in patients who received platinum alone. Thus, across independent data sets taxane-specific association between higher WWOX expression and longer patient OS was observed. As WWOX is often deleted in tumours, we investigated the TCGA data set to understand the impact on survival of WWOX expression being classed as detectable or absent relative to normal physiological levels. Using the median expression across normal tissue samples (eight normal samples in TCGA) to estimate population mean, and the median absolute deviation to estimate the population standard deviation, the 10th percentile of expression (extrapolated from a normal distribution across the normal tissues) was used as a cutoff to reflect present *versus* absent WWOX expression in patient samples. Figures 6e and f show that patients who received Taxol showed longer OS in the presence of WWOX compared with the absence of WWOX (*n*=373, *P*=0.050) and this distinction was lost in the absence of Taxol (*n*=33, *P*=0.92).

#### WWOX expression and PFS

**Figure 7:**
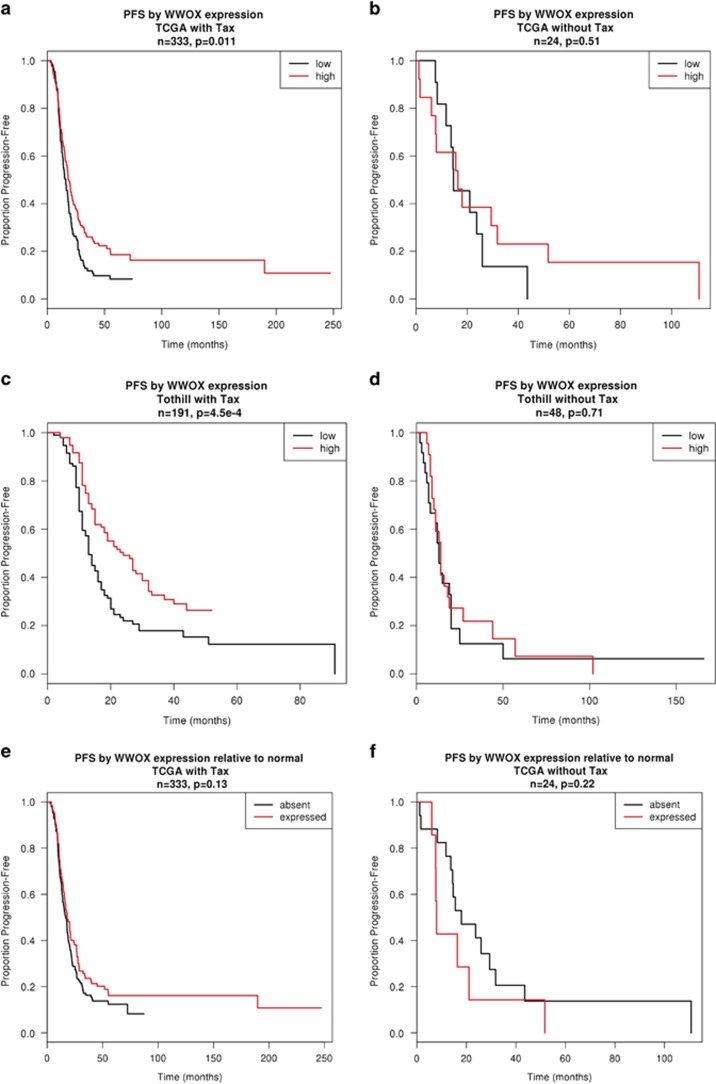
WWOX levels predict PFS in taxane-treated ovarian cancer patients. Kaplan–Meier plots to show the effect of WWOX expression on PFS in two ovarian cancer cohorts namely (**a**) TCGA (*n*=491) and (**b**) Tothill (*n*=258). PFS plots of patients in the TCGA cohort treated with platinum alone (**d**) and taxane+platinum (**c**) and survival plots in the Tothill data set of patients treated with platinum alone (**f**) or taxane+platinum (**e**). Red line represents patients with higher WWOX expression, whereas black line represents patients with lower WWOX expression

We further investigated the association between WWOX expression and progression-free survival (PFS) (time of death or disease progression). Figures 7a–d represent Kaplan–Meier curves of PFS for patients partitioned into WWOX-high (greater than median expression) or WWOX-low. In both TCGA and Tothill cohorts, when patients were treated with platinum and taxane, those with higher WWOX expression had significantly longer PFS than those with lower WWOX (TCGA *n*=333, *P*=0.011; Tothill *n*=191, *P*=0.00045). However, no significant difference in PFS according to WWOX expression was observed in patients who received the platinum alone (Figures 7b and d). We used the TCGA data set to investigate the impact on progression of WWOX expression being classed as detectable or absent relative to normal physiological levels. Corresponding Kaplan–Meier curves in Figures 7e and f show that, in patients treated with platinum and taxane (Figure 7e) but not in patients treated with platinum alone (Figure 7f), this dichotomisation suggests longer PFS in patients with WWOX expression detectable at a level comparable to normal tissue.

#### WWOX expression influences ovarian cancer molecular subtypes

**Figure 8:**
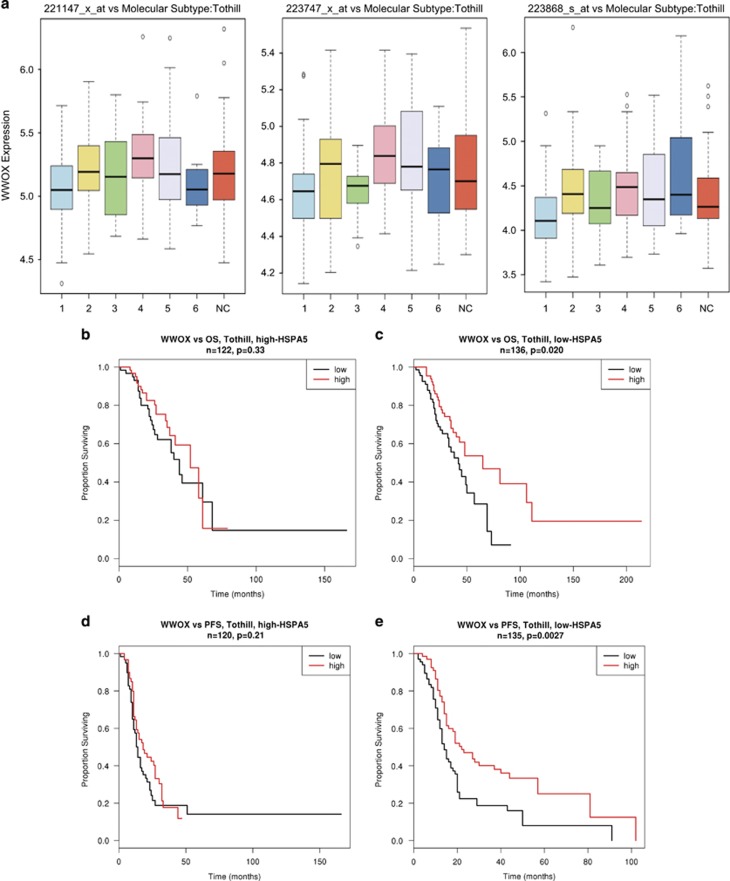
Lower WWOX is characteristic of more aggressive type of ovarian cancer and improved survival through WWOX involves ER stress. (**a**) Box plots to show WWOX expression across three probesets demonstrating in all three that the most aggressive tumour type namely ‘1’ has significantly lower WWOX expression.^31^ Kaplan–Meier plots to show that the effect of WWOX expression on OS (**b** and **c**) and PFS (**d** and **e**) was effectively seen in patient cohorts that had lower endogenous expression of GRP78 (**c** and **e**) and not in those with higher GRP78 expression (**b** and **d**)

WWOX expression showed very significant variation in expression levels across the molecular subtypes reported in the Tothill study.^31^ The poor outcome ovarian cancer subtype ‘1’ had lower WWOX expression across three probesets (Figure 8a, *P*=0.0035; left, *P*<1x10^−4^; middle and *P*=0.00069; right), implying that the loss of WWOX expression has a role in development of aggressive characteristics of this subtype of ovarian cancer.

#### WWOX, associations with ER stress genes and influence on survival

To investigate the clinical relevance of WWOX expression and ER stress genes on survival, we investigated the interaction between a panel of genes transcriptionally upregulated by ER stress (*GRP78, XBP1, ATF6*, eukaryotic translation initiation factor 2-alpha kinase 3 (*EIF2AK3*), *ERN1* and DNA damage-inducible transcript 3) and association of WWOX expression. For each of the panel of genes, proportional hazards regression models were fitted to evaluate the additive effects of WWOX and the ER stress gene, and the interaction between the two genes. It was striking that the association between higher WWOX expression and better outcomes (longer OS and PFS) was dependent on the endogenous levels of GRP78 (Figures 8b–e) but none of the other factors examined (not shown). GRP78 is the best described marker of ER stress, regarded as a key regulator of multiple arms of the UPR, and transcriptionally regulated via conserved ER stress response elements in its promoter.^9^ We found that the impact of WWOX expression on OS (Figures 8b and c) and PFS (Figures 8d and e) was effectively seen in patients (Tothill data set) who had lower endogenous expression of GRP78 (Figures 8c and e) and not in those who had higher GRP78 expression (Figures 8b and d). The patients were split according to GRP78 expression being above or below the median value. The median was 2.42-fold greater than the minimum value and 3.25-fold lower than the maximum value, making the total range of expression (lowest to highest) 7.89-fold. The samples with high GRP78 expression likely represent tumours with intrinsic ER stress leading to apoptosis, irrespective of chemotherapy, and it might be speculated that WWOX status has no impact on these tumours, which have activation of proapoptotic pathways before chemotherapy commences. Also, tumours with high GRP78 may represent evolved tumours with intrinsic ER stress, which is more inclined towards a cytoprotective UPR function. Conversely, WWOX appears to strongly affect PFS in the ‘low GRP78’ tumours, and this may correspond to tumours with intrinsically low, but paclitaxel-inducible, ER stress. These data suggest an inter-relationship between WWOX and the UPR. We could not however detect any interaction between WWOX and GRP78 under the influence of paclitaxel in co-immunoprecipitation assays performed in WWOX-8 cells (Supplementary Figure 9).

## Discussion

The combination of platinum and taxanes is the gold standard for chemotherapy in ovarian cancer; however, cure rates remain low. Our data suggest that WWOX regulates taxane response in ovarian cancer by modulating the drug-related ER stress response. One-third of ovarian cancers exhibit loss of WWOX protein^20^ and these would therefore be expected to respond less to paclitaxel. Turning to the clinical relevance of our findings, WWOX expression levels predicted longer OS and PFS in a population of paclitaxel-treated ovarian cancer patients.

Recently, WWOX has been shown to be responsible for cancer cells' response to chemotherapy on two accounts. WWOX sensitises squamous cell carcinoma cells to apoptosis induced by the antifolate chemotherapeutic agent methotrexate by regulating autophagy responses in cancer cells and absence of WWOX leads to chemotherapeutic drug resistance.^33^ A second study demonstrated that WWOX through its WW1 domain interacts with the p53 homologue protein ΔNP63 known to induce chemoresistance in cancer cells. WWOX sequesters ΔNP63 in the cytoplasm, thus changing its cellular location and transactivation of its target genes. WWOX could reverse ΔNP63-induced chemoresistance to cisplatin by sensitising cancer cells to cisplatin-induced cell death.^34^

ER stress response is also activated in hypoxic or nutrient-deprived tumours,^9, 25, 35, 36, 37, 38^ and thus WWOX loss might be the key to multistep carcinogenesis that enables tumours reaching a macroscopic size to survive under conditions of cellular stress due to stimuli such as hypoxia. WWOX, via its WW domain, physically interacts with hypoxia-inducible factor 1α subunit and modulates its levels and transactivation functions.^39^ Multiple myeloma cells are stressed by unfolded protein overload because of their production of monoclonal immunoglobulins. Interestingly, WWOX is frequently lost or undergoes recurrent genomic alterations in multiple myeloma and this is associated with poor outcomes.^40, 41, 42, 43, 44, 45, 46^ Tumours resembling human multiple myeloma were also noted in WWOX-deficient mice.^12^

Here, through modulating WWOX expression in ovarian cancer cells we show that WWOX influences paclitaxel-induced cell death through ER stress pathway and an intact IRE-1 arm of UPR is crucial for WWOX to function. The bioinformatics data presented in Figure 8 captured the expression levels of GRP78 to be a determinant and an indicator of WWOX-mediated influences on patient survival. GRP78 activates all arms of ER stress including IRE-1 and PERK and its expression along with WWOX could be used as a predictive biomarker of patient responses to paclitaxel treatment. WWOX interacts with SCOTIN/SHISA5,^47^ which was recently demonstrated to be a proapoptotic protein involved in ER stress response.^48, 49^ With two interaction-rich WW domains and an enzymatic tail, WWOX could be involved in protein transport, act as a chaperone or it may regulate redox status, particularly since WWOX knockout mice display severe metabolic abnormalities.^50, 51^ WWOX involvement in ER stress response is consistent with the stress response network hypothesis for common fragile site genes based on the conservation of their loci including the intronic or intragenic fragile site structure that could serve as stress sensors and involvement in stress response already ascribed to WWOX, fragile histidine triad and RAR-related orphan receptor.^52^ In conclusion, our study provides novel insights into the function of the WWOX and suggests that defective proapoptotic signalling during ER stress response associated with loss of WWOX may confer paclitaxel resistance in a proportion of ovarian cancer patients. In general, adaptive UPR mechanisms could be targeted to increase chemotherapy efficacy where that cytotoxic mechanism is mediated by ER stress.

## Materials and methods

### Cell lines

The PEO1 ovarian cancer cell line (derived within our previous laboratory in University of Edinburgh) is homozygously deleted for WWOX exons 4–8 and lacks WWOX protein expression. We developed independent stable WWOX- and vector-transfected lines as described previously.^21, 22^ Cells were maintained in RPMI containing 10% FCS, 100 U/ml penicillin, 100 *μ*g/ml streptomycin (and 3 *μ*g/ml blasticidin for stably transfected cells) and cultured at 37 °C, 5% CO_2_. PEO1 line derivatives were recently authenticated by confirming the presence of a specific homozygous deletion within WWOX by RT-PCR; the presence of plasmid vector by blasticidin selection; and that WWOX was expressed in WWOX-transfected lines, but null in vector-transfected lines by qRT-PCR and immunoblotting.^21, 22^

### Growth curves

Log-phase cells (6 × 10^3^ per well) were seeded into 96-well plates. Following 24 h incubation, media were replaced with media containing paclitaxel, cisplatin, monastrol, tunicamycin, SP600125, KIRA6 or GSK2656157. Monastrol and SP600125 were obtained from Tocris Bioscience (Bristol, England, UK) and tunicamycin from Sigma-Aldrich (St. Louis, MO, USA), KIRA6 from Calbiochem (San Diego, CA, USA) and GSK2656157 from Selleckchem (Houston, TX, USA). Cells were quantified by sulforhodamine B (SRB) assay.

### Caspase-3/7 activation assays

Log-phase cells (1.5 × 10^4^ per well) were seeded into 96-well plates. Following 24 h incubation, media were replaced with media containing paclitaxel. Caspase-3/7 activity was measured after 24 h of drug exposure with CaspaseGlo3/7 Assay Kit (Promega, Fitchburg, WI, USA).

### Annexin V apoptosis assays

Log-phase cells (2.5 × 10^5^ per well) were seeded into 6-well plates. Following 24 h incubation, media were replaced with media containing paclitaxel. After 24 h of drug exposure, floating and adherent cells were collected and Annexin V measured by flow cytometry using TACSTM Annexin V-FITC Apoptosis Detection Kit (R&D Systems, Minneapolis, MN, USA).

### Measuring mitotic index

Cells were plated and treated as for Annexin V assays. Following drug exposure, the cells were fixed with 2% paraformaldehyde, blocked with 1% BSA and permeabilized with 1 × BDPerm/Wash solution (BD Biosciences, San Jose, CA, USA). Cells (2 × 10^5^) were stained with 1:100 dilution of anti-MPM2 antibody (Millipore, Leicester, UK), followed by incubation with 1:500 dilution of secondary fluorescein isothiocyanate (FITC)-conjugated antibody (Sigma-Aldrich). Cells were analysed by fluorescence-activated cell sorting (FACS) and the percentage of MPM2-positive cells was designated as mitotic index.

### RNAi transfections

A total of 2.5 × 10^5^ cells were incubated in 6-well plate for 24 h. One hundred microlitres of 2 *μ*M ITGA3 siRNA SMARTpool, WWOX siRNA SMARTpool (Dharmacon, Lafayette, CO, USA), oligo 1, 15 or non-targeting oligonucleotide pool 2 (Perbio, Helsingborg, Skane Lan, Sweden) were mixed with 100 *μ*l of Optimem media and incubated for 5 min. Three microlitres of Dharmafect transfection reagent 1 (Perbio) was incubated with 197 *μ*l Optimem media for 5 min, combined with relevant siRNA solution for 20 min, mixed with medium and added to cells. To measure the level of ITGA3 knockdown, cells were collected 72 h post transfection, fixed with 2% paraformaldehyde and blocked with 1% BSA. Cells (2 × 10^5^) were stained with 1:100 dilution of ITGA3 antibody (Millipore), followed by incubation with 1:500 dilution of secondary FITC-conjugated antibody (Sigma) and analysed by FACS.

### Quantitative RT-PCR

QRT-PCR was conducted as described previously.^19^ Primer sequences: TGFBI (5′-ATGGGGACTGTCATGGATGT-3′ and 5′-TGTAGACTCCTTCCCGGTTG-3′), PPIA (5′-CTGCACTGCCAAGACTGA-5′ and 5′-GCCATTCCTGGACCCAAA-3′), TUBA1B (5′-GCCAAGCGTGCCTTTGTTC-3′ and 5′-CACACCAACCTCCTCATAATCC-3′).

### Protein arrays

Cells were plated and treated as for Annexin V assays. Cell lysates from three experiments (100 *μ*g protein each) were combined, incubated with human apoptosis protein or phosphoprotein arrays (ARY009 and ARY003; R&D Systems) and processed as per the manufacturer’s protocol. The films were scanned with transmission-mode scanner and pixel densities analyzed with ImageQuant (GE Healthcare, Chicago, IL, USA).

### Western blotting

Western blotting was conducted according to standard protocols. The antibodies used in this study were as follows: rabbit anti-WWOX (a kind gift of Dr. M Aldaz, MD Anderson Cancer Centre, Houston, TX, USA) and ab137726 antibody (abcam), rabbit anti-*β*-actin (no. 4970; Cell Signalling Technology, Danvers, MA, USA), rabbit anti-Hsp60 (abcam; ab46798), rabbit anti-cleaved (Asp175) caspase-3 (R&D Systems, Minneapolis, MN, USA; 269518), mouse caspase-2 (no. 2224; Cell Signalling Technology), rabbit caspase-7 (no. 9492; Cell Signalling Technology), rabbit anti-phospho-eIF2A (Cell Signalling Technology; 9721), mouse anti-GRP78 (474421; R&D Systems), mouse anti-JNK1/JNK2 252323, MAB2076 and rabbit AF1205 (phospho-JNK) (R&D Systems), rabbit anti-phospho (T183/Y185)-JNK (AF1205), rabbit IRE-1 (ab48187 and ab37073; abcam) and PERK (no. 3192; Cell Signalling Technology).

### Co-immunoprecipitation

The cells were cultured on 15-cm Petri dishes and collected when they reached 70–80% confluence (when using PEO1 stable transfectants the medium with blasticidin was replaced with media without the drug 24 h before plating for the experiment). The cells were washed with ice-cold PBS, and detached with a cell scraper into 5 ml volume of fresh PBS, centrifuged and lysed immediately or stored as a pellet in −80 °C. Cells collected from each dish were lysed in 400 *μ*l of the following buffer for 30 min on ice (0.5–1% Triton X or 0.5–1% NP40, 10% glycerol, 150 mM NaCl, 50 mM Tris-HCl (pH=7.2), 0.2 mM Na_3_VO_4_, 50 mM NaF, 2 mM EDTA, 1 mM PMSF, supplemented with 100 U/ml aprotinin, 10 *μ*g/ml leupeptin and 1 *μ*g/ml pepstatin). The lysates were centrifuged at 13 000 r.p.m. at 4 °C for 20 min and the supernatant was transferred to a new tube. The supernatant in each tube was precleared by incubation for 1 h with 10 *μ*l of Protein A/G UltraLink Resin (Pierce, Waltham, MA, USA; no. 53132). Two micrograms of a relevant or control antibody as appropriate was added to each precleared sample and incubated at 4 °C on a circular shaker for 3–6 h. Subsequently, 10 *μ*l of the resin was added to each sample and incubated at 4 °C on a circular shaker for 3 h to overnight. The beads pellets were washed four times with the lysis buffer and resuspended in 30 *μ*l of 1xPAGE loading buffer (Fermentas, Waltham, MA, USA; no. R0611), 76 supplemented with DTT to the final concentration of 100 mM and boiled at 95 °C for 5 min. The immunoprecipitates were analysed by immunoblotting as described above.

### Bioinformatics and statistical analysis

The TCGA data set, featuring over 500 primary tumour samples, was obtained from The Cancer Genome Atlas (https://tcga-data.nci.nih.gov/tcga/). The ‘Tothill’ data set featuring nearly 300 primary tumour samples was obtained from Gene Expression Omnibus (series accession GSE9891). Raw gene expression data (Affymetrix CEL files) were obtained for each data set and normalised using RMA.^32^ Statistics derived from Cox proportional hazards models were used to evaluate the quantitative association between a unit increase of some measure of term(s) of interest and either PFS time or OS time. Dichotomisation of patient cohorts into higher-than-median expression of a gene of interest and lower-than-median expression of the gene of interest was carried out for the purposes of illustrating effects in Kaplan–Meier plots. Statistical significance of differences in survival curves were evaluated using log-rank tests. Linear models were fitted with LIMMA to derive F-statistics to assess the variation of a given probe’s measured gene expression levels across samples grouped according to the categorical clinical variables ‘residual disease following primary surgery’ and ‘molecular subtype’.

## References

[bib1] Vaughan S, Coward JI, Bast RC Jr, Berchuck A, Berek JS, Brenton JD et al. Rethinking ovarian cancer: recommendations for improving outcomes. Nat Rev Cancer 2011; 11: 719–725. 21941283 10.1038/nrc3144 PMC3380637

[bib2] Jordan MA, Wilson L. Microtubules as a target for anticancer drugs. Nat Rev Cancer 2004; 4: 253–265. 15057285 10.1038/nrc1317

[bib3] Shi J, Orth JD, Mitchison T. Cell type variation in responses to antimitotic drugs that target microtubules and kinesin-5. Cancer Res 2008; 68: 3269–3276. 18451153 10.1158/0008-5472.CAN-07-6699

[bib4] Swanton C, Marani M, Pardo O, Warne PH, Kelly G, Sahai E et al. Regulators of mitotic arrest and ceramide metabolism are determinants of sensitivity to paclitaxel and other chemotherapeutic drugs. Cancer Cell 2007; 11: 498–512. 17560332 10.1016/j.ccr.2007.04.011

[bib5] Liao PC, Tan SK, Lieu CH, Jung HK. Involvement of endoplasmic reticulum in paclitaxel-induced apoptosis. J Cell Biochem 2008; 104: 1509–1523. 18452161 10.1002/jcb.21730

[bib6] Mhaidat NM, Alali FQ, Matalqah SM, Matalka II, Jaradat SA, Al-Sawalha NA et al. Inhibition of MEK sensitizes paclitaxel-induced apoptosis of human colorectal cancer cells by downregulation of GRP78. Anticancer Drugs 2009; 20: 601–606. 19521235 10.1097/CAD.0b013e32832e3120

[bib7] Boehmerle W, Splittgerber U, Lazarus MB, McKenzie KM, Johnston DG, Austin DJ et al. Paclitaxel induces calcium oscillations via an inositol 1,4,5-trisphosphate receptor and neuronal calcium sensor 1-dependent mechanism. Proc Natl Acad Sci USA 2006; 103: 18356–18361. 17114292 10.1073/pnas.0607240103 PMC1838755

[bib8] Mhaidat NM, Thorne R, Zhang XD, Hersey P. Involvement of endoplasmic reticulum stress in docetaxel-induced JNK-dependent apoptosis of human melanoma. Apoptosis 2008; 13: 1505–1512. 18989785 10.1007/s10495-008-0276-8

[bib9] Kim I, Xu W, Reed JC. Cell death and endoplasmic reticulum stress: disease relevance and therapeutic opportunities. Nat Rev Drug Discov 2008; 7: 1013–1030. 19043451 10.1038/nrd2755

[bib10] Bednarek AK, Laflin KJ, Daniel RL, Liao Q, Hawkins KA, Aldaz CM. WWOX, a novel WW domain-containing protein mapping to human chromosome 16q23.3-24.1, a region frequently affected in breast cancer. Cancer Res 2000; 60: 2140–2145. 10786676

[bib11] Aqeilan RI, Trapasso F, Hussain S, Costinean S, Marshall D, Pekarsky Y et al. Targeted deletion of Wwox reveals a tumor suppressor function. Proc Natl Acad Sci USA 2007; 104: 3949–3954. 17360458 10.1073/pnas.0609783104 PMC1820689

[bib12] Ludes-Meyers JH, Kil H, Nunez MI, Conti CJ, Parker-Thornburg J, Bedford MT et al. WWOX hypomorphic mice display a higher incidence of B-cell lymphomas and develop testicular atrophy. Genes Chromosomes Cancer 2007; 46: 1129–1136. 17823927 10.1002/gcc.20497 PMC4143238

[bib13] Abdeen SK, Salah Z, Maly B, Smith Y, Tufail R, Abu-Odeh M et al. Wwox inactivation enhances mammary tumorigenesis. Oncogene 2011; 30: 3900–3906. 21499303 10.1038/onc.2011.115

[bib14] Aqeilan RI, Croce CM. WWOX in biological control and tumorigenesis. J Cell Physiol 2007; 212: 307–310. 17458891 10.1002/jcp.21099

[bib15] Dias EP, Pimenta FJ, Sarquis MS, Dias Filho MA, Aldaz CM, Fujii JB et al. Association between decreased WWOX protein expression and thyroid cancer development. Thyroid 2007; 17: 1055–1059. 18047428 10.1089/thy.2007.0232 PMC4150466

[bib16] Chang NS, Hsu LJ, Lin YS, Lai FJ, Sheu HM. WW domain-containing oxidoreductase: a candidate tumor suppressor. Trends Mol Med 2007; 13: 12–22. 17142102 10.1016/j.molmed.2006.11.006

[bib17] Yang J, Cogdell D, Yang D, Hu L, Li H, Zheng H et al. Deletion of the WWOX gene and frequent loss of its protein expression in human osteosarcoma. Cancer Lett 2010; 291: 31–38. 19896763 10.1016/j.canlet.2009.09.018

[bib18] Aldaz CM, Ferguson BW, Abba MC. WWOX at the crossroads of cancer, metabolic syndrome related traits and CNS pathologies. Biochim Biophys Acta 2014; 1846: 188–200. 24932569 10.1016/j.bbcan.2014.06.001 PMC4151823

[bib19] Chang HT, Liu CC, Chen ST, Yap YV, Chang NS, Sze CI. WW domain-containing oxidoreductase in neuronal injury and neurological diseases. Oncotarget 2014; 5: 11792–11799. 25537520 10.18632/oncotarget.2961 PMC4322972

[bib20] Nunez MI, Rosen DG, Ludes-Meyers JH, Abba MC, Kil H, Page R et al. WWOX protein expression varies among ovarian carcinoma histotypes and correlates with less favorable outcome. BMC Cancer 2005; 5: 64. 15982416 10.1186/1471-2407-5-64 PMC1173095

[bib21] Gourley C, Paige AJ, Taylor KJ, Ward C, Kuske B, Zhang J et al. WWOX gene expression abolishes ovarian cancer tumorigenicity *in vivo* and decreases attachment to fibronectin via integrin alpha3. Cancer Res 2009; 69: 4835–4842. 19458077 10.1158/0008-5472.CAN-08-2974

[bib22] Gourley C, Paige AJ, Taylor KJ, Scott D, Francis NJ, Rush R et al. WWOX mRNA expression profile in epithelial ovarian cancer supports the role of WWOX variant 1 as a tumour suppressor, although the role of variant 4 remains unclear. Int J Oncol 2005; 26: 1681–1689. 15870886 10.3892/ijo.26.6.1681 PMC4166600

[bib23] Mayer TU, Kapoor TM, Haggarty SJ, King RW, Schreiber SL, Mitchison TJ. Small molecule inhibitor of mitotic spindle bipolarity identified in a phenotype-based screen. Science 1999; 286: 971–974. 10542155 10.1126/science.286.5441.971

[bib24] Ahmed AA, Mills AD, Ibrahim AE, Temple J, Blenkiron C, Vias M et al. The extracellular matrix protein TGFBI induces microtubule stabilization and sensitizes ovarian cancers to paclitaxel. Cancer Cell 2007; 12: 514–527. 18068629 10.1016/j.ccr.2007.11.014 PMC2148463

[bib25] Ferri KF, Kroemer G. Organelle-specific initiation of cell death pathways. Nat Cell Biol 2001; 3: E255–E263. 11715037 10.1038/ncb1101-e255

[bib26] Chang NS, Doherty J, Ensign A. JNK1 physically interacts with WW domain-containing oxidoreductase (WOX1) and inhibits WOX1-mediated apoptosis. J Biol Chem 2003; 278: 9195–9202. 12514174 10.1074/jbc.M208373200

[bib27] Bennett BL, Sasaki DT, Murray BW, O'Leary EC, Sakata ST, Xu W et al. SP600125, an anthrapyrazolone inhibitor of Jun N-terminal kinase. Proc Natl Acad Sci USA 2001; 98: 13681–13686. 11717429 10.1073/pnas.251194298 PMC61101

[bib28] Harding HP, Zhang Y, Ron D. Protein translation and folding are coupled by an endoplasmic-reticulum-resident kinase. Nature 1999; 397: 271–274. 9930704 10.1038/16729

[bib29] Novoa I, Zhang Y, Zeng H, Jungreis R, Harding HP, Ron D. Stress-induced gene expression requires programmed recovery from translational repression. EMBO J 2003; 22: 1180–1187. 12606582 10.1093/emboj/cdg112 PMC150345

[bib30] Cancer Genome Atlas Research N. Integrated genomic analyses of ovarian carcinoma. Nature 2011; 474: 609–615. 21720365 10.1038/nature10166 PMC3163504

[bib31] Tothill RW, Tinker AV, George J, Brown R, Fox SB, Lade S et al. Novel molecular subtypes of serous and endometrioid ovarian cancer linked to clinical outcome. Clin Cancer Res 2008; 14: 5198–5208. 18698038 10.1158/1078-0432.CCR-08-0196

[bib32] Irizarry RA, Hobbs B, Collin F, Beazer-Barclay YD, Antonellis KJ, Scherf U et al. Exploration, normalization, and summaries of high density oligonucleotide array probe level data. Biostatistics 2003; 4: 249–264. 12925520 10.1093/biostatistics/4.2.249

[bib33] Tsai CW, Lai FJ, Sheu HM, Lin YS, Chang TH, Jan MS et al. WWOX suppresses autophagy for inducing apoptosis in methotrexate-treated human squamous cell carcinoma. Cell Death Dis 2013; 4: e792. 24008736 10.1038/cddis.2013.308 PMC3789168

[bib34] Salah Z, Bar-mag T, Kohn Y, Pichiorri F, Palumbo T, Melino G et al. Tumor suppressor WWOX binds to DeltaNp63alpha and sensitizes cancer cells to chemotherapy. Cell Death Dis 2013; 4: e480. 23370280 10.1038/cddis.2013.6 PMC3564006

[bib35] Feldman DE, Chauhan V, Koong AC. The unfolded protein response: a novel component of the hypoxic stress response in tumors. Mol Cancer Res 2005; 3: 597–605. 16317085 10.1158/1541-7786.MCR-05-0221

[bib36] Rao RV, Ellerby HM, Bredesen DE. Coupling endoplasmic reticulum stress to the cell death program. Cell Death Differ 2004; 11: 372–380. 14765132 10.1038/sj.cdd.4401378

[bib37] Ma Y, Hendershot LM. The role of the unfolded protein response in tumour development: friend or foe? Nat Rev Cancer 2004; 4: 966–977. 15573118 10.1038/nrc1505

[bib38] Healy SJ, Gorman AM, Mousavi-Shafaei P, Gupta S, Samali A. Targeting the endoplasmic reticulum-stress response as an anticancer strategy. Eur J Pharmacol 2009; 625: 234–246. 19835867 10.1016/j.ejphar.2009.06.064

[bib39] Abu-Remaileh M, Aqeilan RI. Tumor suppressor WWOX regulates glucose metabolism via HIF1alpha modulation. Cell Death Differ 2014; 21: 1805–1814. 25012504 10.1038/cdd.2014.95 PMC4211377

[bib40] Nagoshi H, Taki T, Hanamura I, Nitta M, Otsuki T, Nishida K et al. Frequent PVT1 rearrangement and novel chimeric genes PVT1-NBEA and PVT1-WWOX occur in multiple myeloma with 8q24 abnormality. Cancer Res 2012; 72: 4954–4962. 22869583 10.1158/0008-5472.CAN-12-0213

[bib41] Chesi M, Bergsagel PL, Shonukan OO, Martelli ML, Brents LA, Chen T et al. Frequent dysregulation of the c-maf proto-oncogene at 16q23 by translocation to an Ig locus in multiple myeloma. Blood 1998; 91: 4457–4463. 9616139

[bib42] Krummel KA, Roberts LR, Kawakami M, Glover TW, Smith DI. The characterization of the common fragile site FRA16D and its involvement in multiple myeloma translocations. Genomics 2000; 69: 37–46. 11013073 10.1006/geno.2000.6321

[bib43] Jenner MW, Leone PE, Walker BA, Ross FM, Johnson DC, Gonzalez D et al. Gene mapping and expression analysis of 16q loss of heterozygosity identifies WWOX and CYLD as being important in determining clinical outcome in multiple myeloma. Blood 2007; 110: 3291–3300. 17609426 10.1182/blood-2007-02-075069

[bib44] Walker BA, Leone PE, Jenner MW, Li C, Gonzalez D, Johnson DC et al. Integration of global SNP-based mapping and expression arrays reveals key regions, mechanisms, and genes important in the pathogenesis of multiple myeloma. Blood 2006; 108: 1733–1743. 16705090 10.1182/blood-2006-02-005496

[bib45] Agnelli L, Mosca L, Fabris S, Lionetti M, Andronache A, Kwee I et al. A SNP microarray and FISH-based procedure to detect allelic imbalances in multiple myeloma: an integrated genomics approach reveals a wide gene dosage effect. Genes Chromosomes Cancer 2009; 48: 603–614. 19396863 10.1002/gcc.20668

[bib46] Zhan F, Hardin J, Kordsmeier B, Bumm K, Zheng M, Tian E et al. Global gene expression profiling of multiple myeloma, monoclonal gammopathy of undetermined significance, and normal bone marrow plasma cells. Blood 2002; 99: 1745–1757. 11861292 10.1182/blood.v99.5.1745

[bib47] Ludes-Meyers JH, Kil H, Bednarek AK, Drake J, Bedford MT, Aldaz CM. WWOX binds the specific proline-rich ligand PPXY: identification of candidate interacting proteins. Oncogene 2004; 23: 5049–5055. 15064722 10.1038/sj.onc.1207680 PMC4143251

[bib48] Bourdon JC, Renzing J, Robertson PL, Fernandes KN, Lane DP. Scotin, a novel p53-inducible proapoptotic protein located in the ER and the nuclear membrane. J Cell Biol 2002; 158: 235–246. 12135983 10.1083/jcb.200203006 PMC2173124

[bib49] Terrinoni A, Ranalli M, Cadot B, Leta A, Bagetta G, Vousden KH et al. p73-alpha is capable of inducing scotin and ER stress. Oncogene 2004; 23: 3721–3725. 15116103 10.1038/sj.onc.1207342

[bib50] Aqeilan RI, Hassan MQ, de Bruin A, Hagan JP, Volinia S, Palumbo T et al. The WWOX tumor suppressor is essential for postnatal survival and normal bone metabolism. J Biol Chem 2008; 283: 21629–21639. 18487609 10.1074/jbc.M800855200 PMC2490770

[bib51] Ludes-Meyers JH, Kil H, Parker-Thornburg J, Kusewitt DF, Bedford MT, Aldaz CM. Generation and characterization of mice carrying a conditional allele of the Wwox tumor suppressor gene. PLoS ONE 2009; 4: e7775. 19936220 10.1371/journal.pone.0007775 PMC2777388

[bib52] Smith DI, McAvoy S, Zhu Y, Perez DS. Large common fragile site genes and cancer. Semin Cancer Biol 2007; 17: 31–41. 17140807 10.1016/j.semcancer.2006.10.003

