# Supplementary material for: WWOX sensitises ovarian cancer cells to paclitaxel via modulation of the ER stress response
Source: Cell Death Dis. 2017 Jul 27;8(7):e2955–. doi: 10.1038/cddis.2017.346 (PMC5550887; doi:10.1038/cddis.2017.346)

Supplementary Figure 1

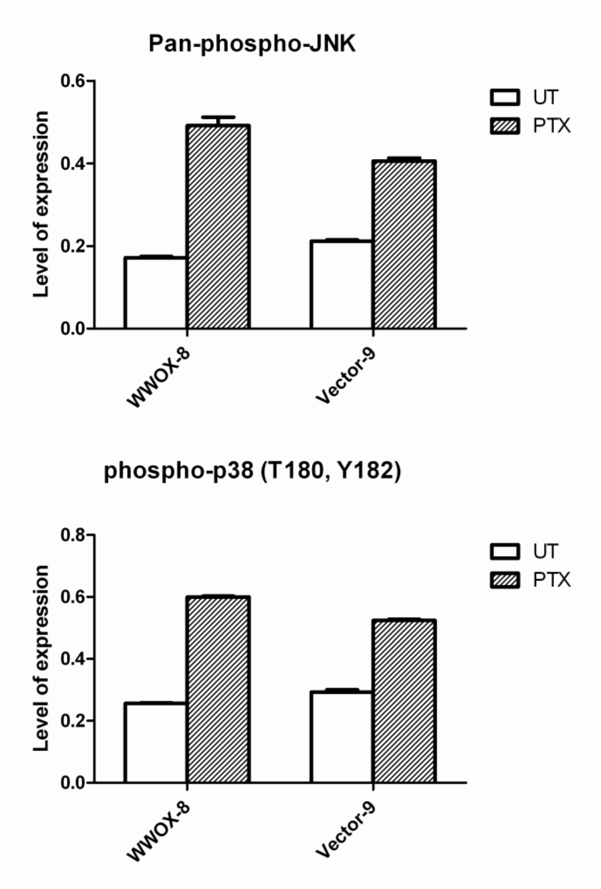

Supplementary Figure 2

A.

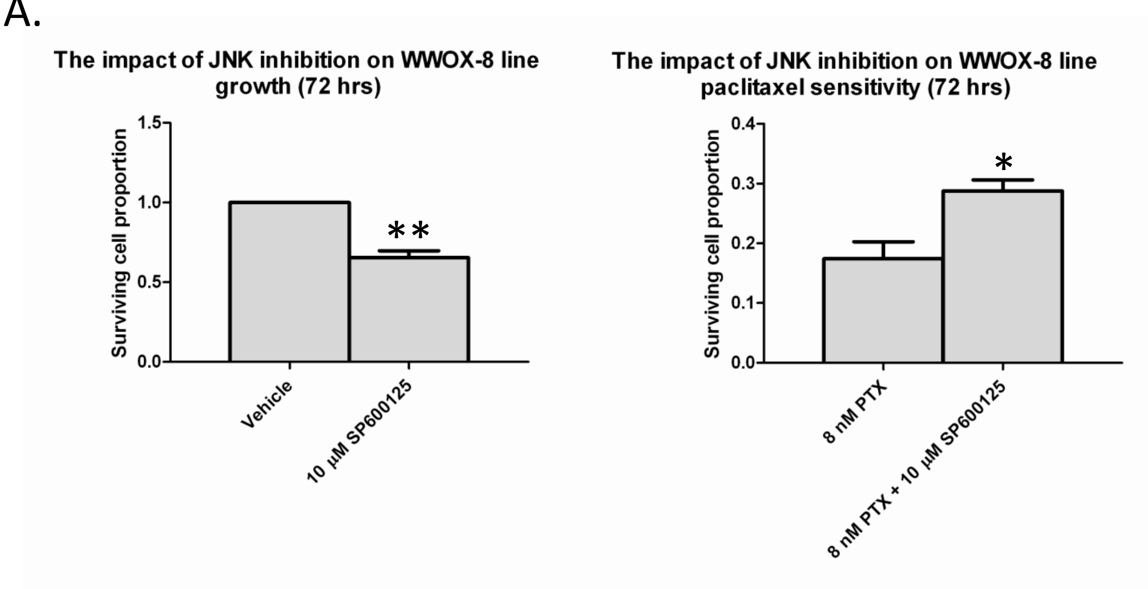

B.

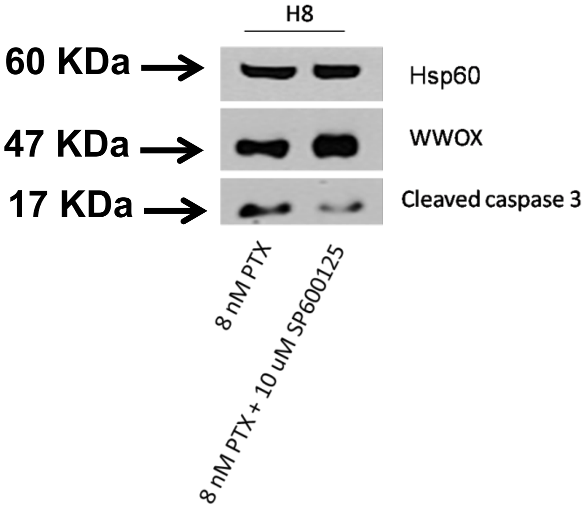

Supplementary Figure 3

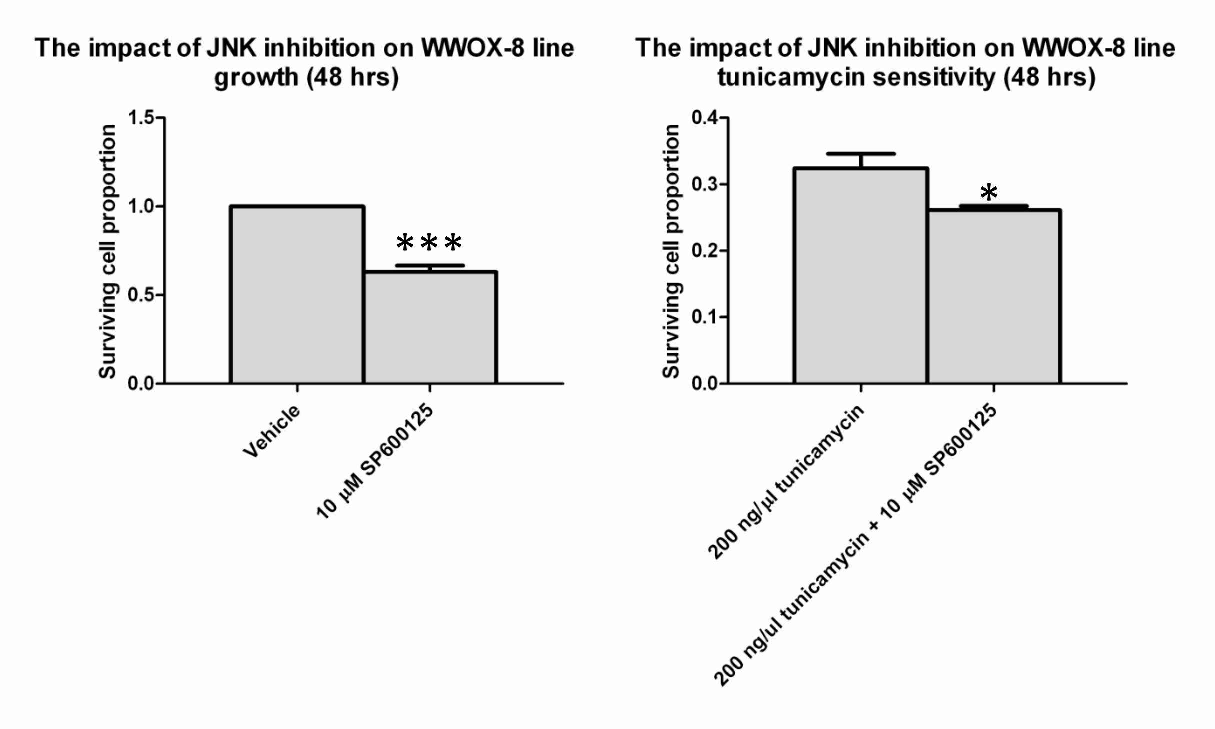

Supplementary Figure 4

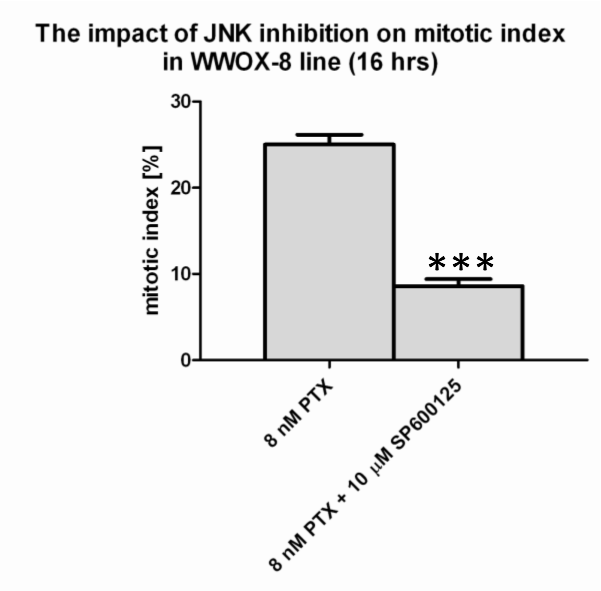

Supplementary Figure 5

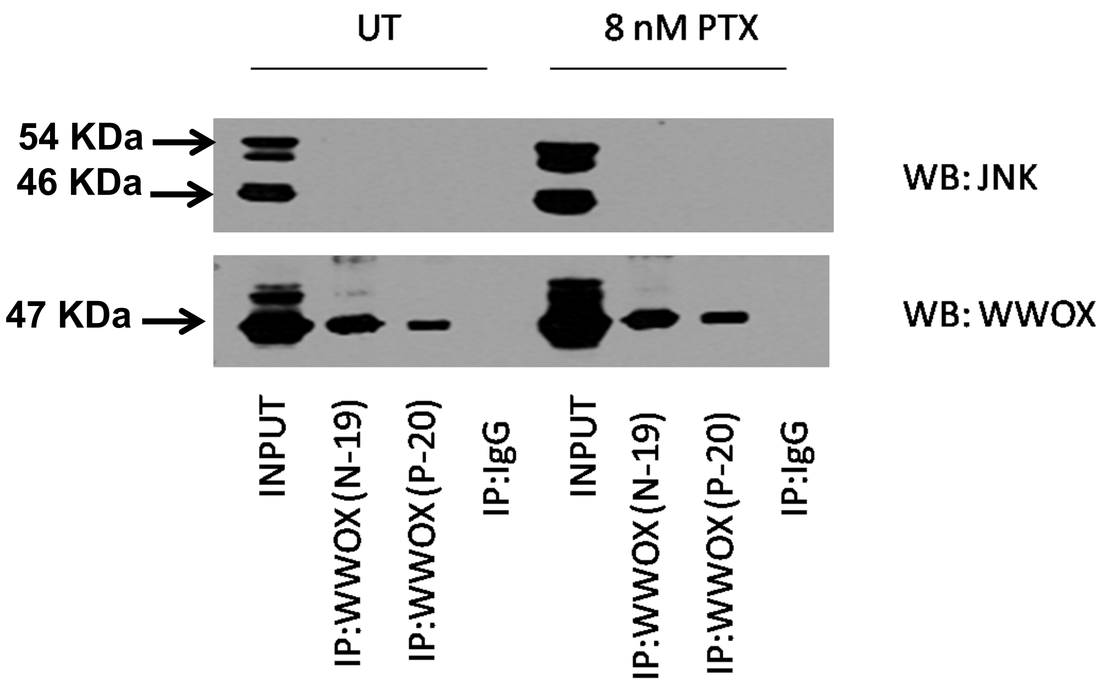

16 hrs 8 nM paclitaxel stimulation

Supplementary Figure 6

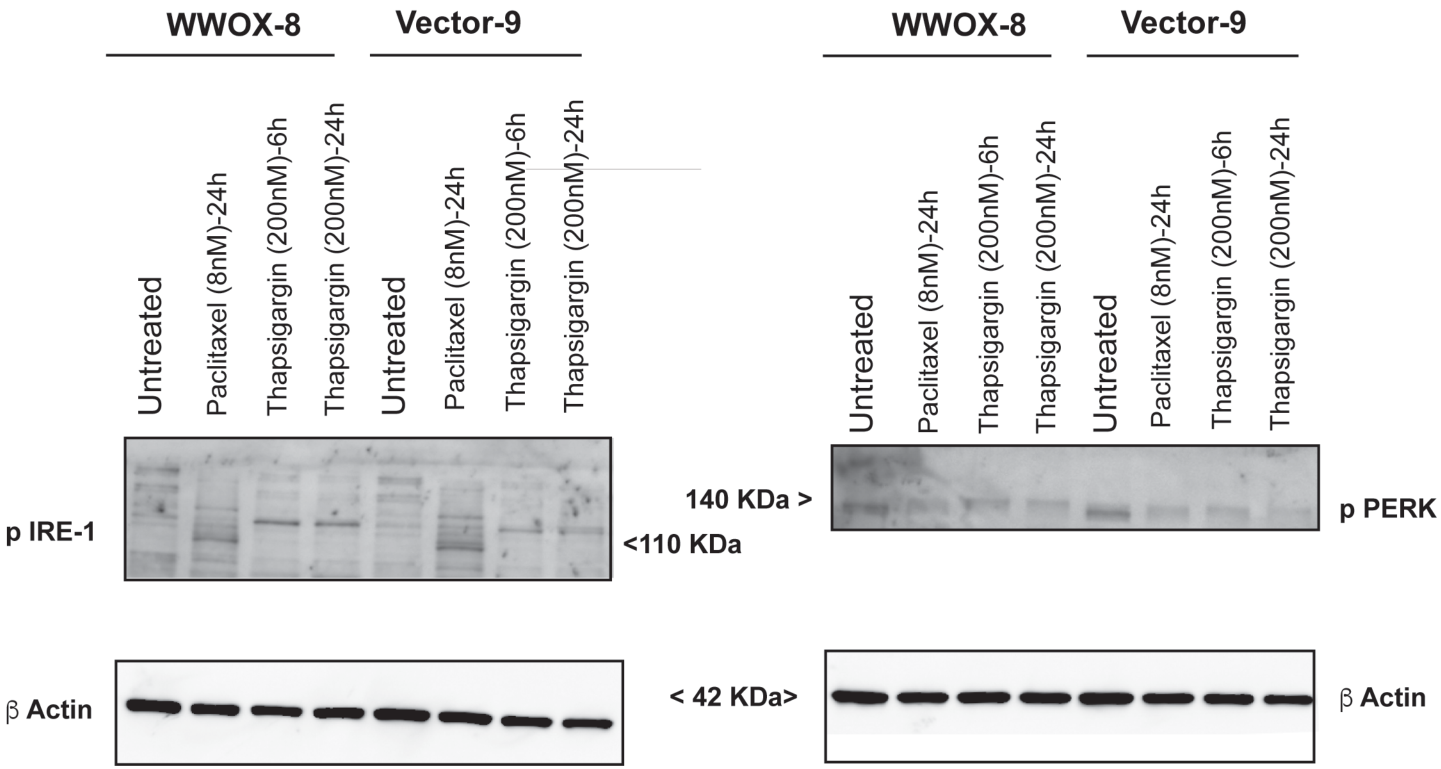

Supplementary Figure 7

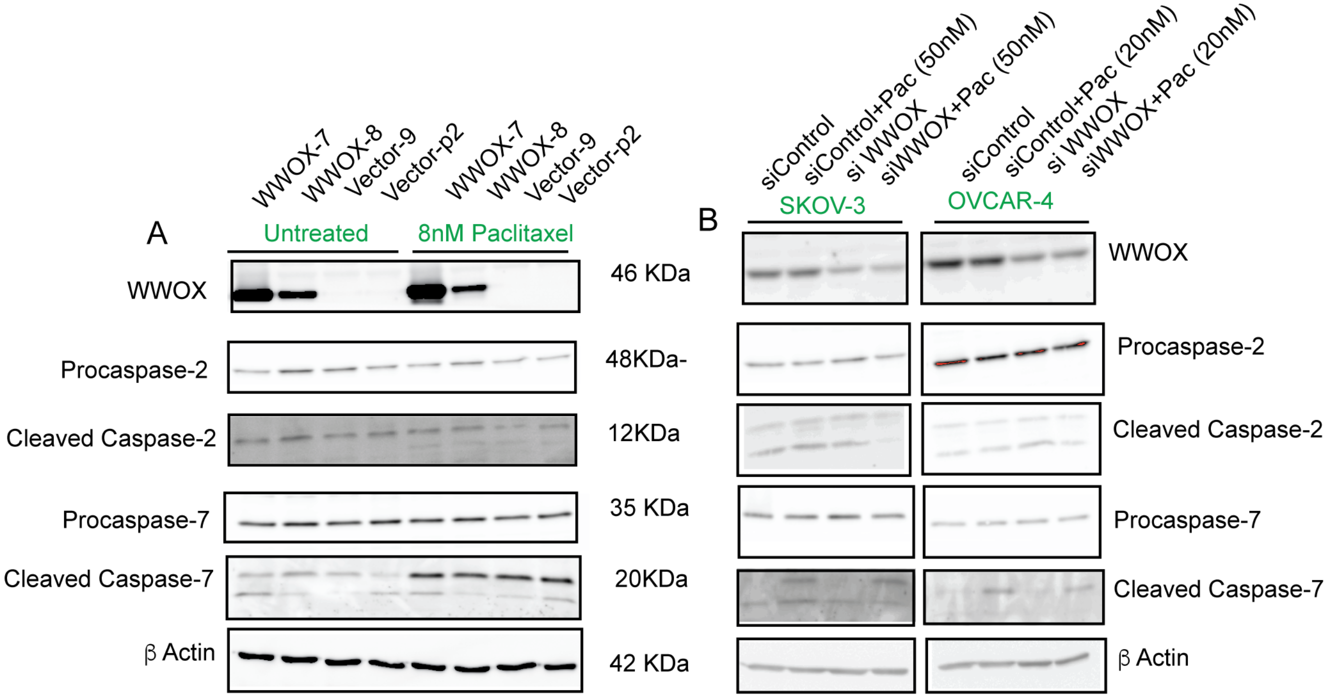

Supplementary Figure 8

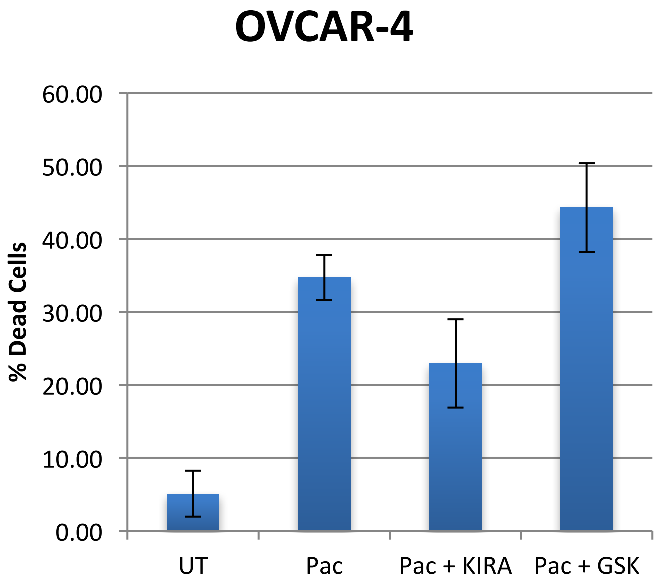

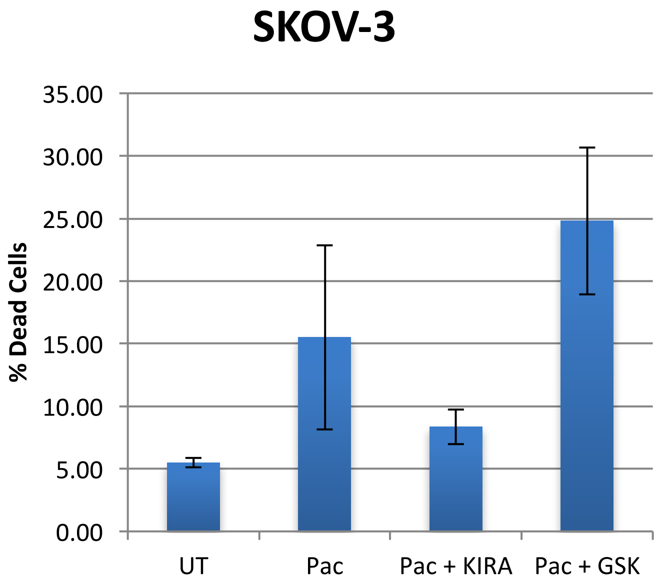

Supplementary Figure 9

Co-immunoprecipitation to check interaction between GRP78 and WWOX

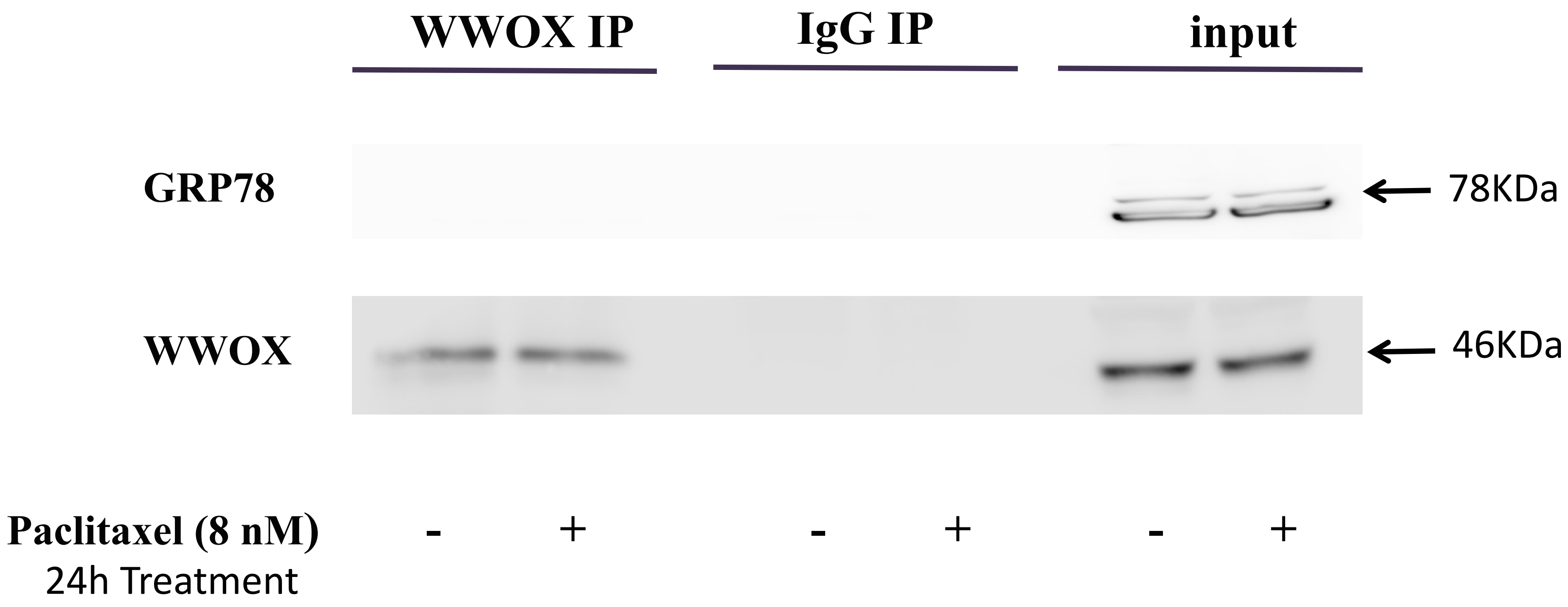

Supplement: Supplementary Figure 1 [file cddis2017346x1.pdf]
