# Supplementary material for: WWOX sensitises ovarian cancer cells to paclitaxel via modulation of the ER stress response
Source: Cell Death Dis. 2017 Jul 27;8(7):e2955–. doi: 10.1038/cddis.2017.346 (PMC5550887; doi:10.1038/cddis.2017.346)
Supplement: Supplementary Figure Legends [file cddis2017346x2.docx]

**Supplementary figure 1.** JNK and p38 mitogen associated protein kinases activation following paclitaxel exposure in PEO1 cells. Densitometry from phosphoprotein arrays. Means of two probe spots ± SD.

**Supplementary figure 2**. JNK inhibition rescues WWOX-8 cells from paclitaxel dependent apoptosis. A, Relative cell survival following 72 hrs SP600125 treatment as compared to vehicle (left) and following 72 hours paclitaxel exposure with or without SP600125 treatment (right), measured by SRB assay. Means of 5 replicates ± SEM. B, Immunoblot showing activated caspase-3 levels (MAB835 antibody, R&D Systems) following 24 hours paclitaxel with or without SP600125 treatment. Hsp60 bands show equal loading of blot. (*, P<0.05; **, P<0.01)

**Supplementary figure 3.** JNK inhibition does not rescue WWOX-8 cells from tunicamycin cytotoxicity. Left panel: Relative cell survival following 72 hours SP600125 exposure as compared to vehicle, measured by SRB assay. Means of 5 replicates ± SEM. Right panel: Relative cell survival following 72 hours tunicamycin treatment, with or without SP600125, measured by SRB assay. Means of 5 replicates ± SEM. (*, P<0.05; ***, P<0.005)

**Supplementary figure 4.** JNK inhibition in WWOX-8 cells antagonizes the anti-mitotic action of paclitaxel. FACS positivity for MPM2 following paclitaxel treatment, with or without SP600125. Means of triplicate experiments ± SEM.

**Supplementary figure 5.** No evidence for WWOX/JNK interaction in WWOX-7 cells. WWOX-7 cells were treated with 8 nM paclitaxel. Cellular lysates were immunoprecipitated with WWOX antibodies and probed for WWOX and JNK1/2.

**Supplementary Figure 6:** PEO1 clones treated with 8nM Paclitaxel and 200μM Thapsigargin (6hr and 24hr) and protein extracts probed with p-IRE-1 (ab48187) and PERK (ab65152) antibodies. β Actin is used as a loading control.

**Supplementary figure 7.** Caspase 2 and 7 activation in paclitaxel treated conditions in the presence of absence of WWOX in ovarian cancer cell lines. Western blots of proteins extracted from (A) untreated or 8nM Paclitaxel treated (24 hour) PEO1 cell lines in the presence or absence of WWOX. (B) SKOV-3 and OVCAR-4 cells, 72hrs after treatment with siWWOX or non targeting control and 24hrs after treatment with paclitaxel at concentration 50nM (SKOV-3) and 20nM (OVCAR-4). Expression of procaspase and cleaved caspase 2,7 was analysed. Caspase 2 and 7(No.2224 and 9492 Cell Signalling Technology antibodies) in ovarian cancer cells. B Actin was used as a loading control.

**Supplementary figure 8.** FACS counts of dead OVCAR-4 and SKOV-3 following Propidium Iodide (PI) staining. Cells were treated with IRE-1 inhibitor (KIRA6; 532281 Calbiochem) and PERK (GSK2656157; S7033 Selleckchem) alone or together in combination with paclitaxel. Cell were treated with 1μg/μl inhibitors, 2 hrs prior to paclitaxel treatment for 24 hours (OVCAR-4 (20nM) and SKOV-3(50nM). Triplicate experiments were performed.

**Supplementary figure 9.** No evidence for WWOX/GRP78 interaction in WWOX-8 cells. WWOX-8 cells were treated with 8 nM paclitaxel. Cellular lysates were immunoprecipitated with WWOX antibodies and probed for WWOX and GRP78
